# What underlies the difference between self-reported health and disability after stroke? A qualitative study in the UK

**DOI:** 10.1186/s12883-021-02338-x

**Published:** 2021-08-13

**Authors:** Nahal Mavaddat, Euan Sadler, Lisa Lim, Kate Williams, Elizabeth Warburton, Ann Louise Kinmonth, Chris Mckevitt, Jonathan Mant

**Affiliations:** 1 School of Medicine, Division of General Practice, University of Western Australia, 35 Stirling Highway, Crawley, Perth, WA, 6009 Australia; 2 Department of Nursing, Midwifery and Health, School of Health Sciences, Faculty of Environmental and Life Sciences, University of Southampton, Southampton, UK; 3 Department of Public Health and Primary Care, University of Cambridge, Strangeways Laboratory, 2 Worts Causeway, Cambridge, CB1 8RN UK; 4 Department of Clinical Neurosciences, Neurology Unit, University of Cambridge, R3, Box 83, Cambridge Biomedical Campus, Cambridge, CB2 0QQ UK; 5 School of Population Health and Environmental Sciences, King’s College London, Addison House, London, SE1 1UL UK

**Keywords:** Stroke self-reported health quality of life disability

## Abstract

**Background:**

Levels of self-reported health do not always correlate with levels of physical disability in stroke survivors. We aimed to explore what underlies the difference between subjective self-reported health and objectively measured disability among stroke survivors.

**Methods:**

Face to face semi-structured interviews were conducted with stroke survivors recruited from a stroke clinic or rehabilitation ward in the UK. Fifteen stroke survivors purposively sampled from the clinic who had discordant self-rated health and levels of disability i.e. reported health as ‘excellent’ or ‘good’ despite significant physical disability (eight), or as ‘fair’ or ‘poor’ despite minimal disability (seven) were compared to each other, and to a control group of 13 stroke survivors with concordant self-rated health and disability levels. Interviews were conducted 4 to 6 months after stroke and data analysed using the constant comparative method informed by Albrecht and Devlieger’s concept of ‘disability paradox’.

**Results:**

Individuals with ‘excellent’ or ‘good’ self-rated health reported a sense of self-reliance and control over their bodies, focussed on their physical rehabilitation and lifestyle changes and reported few bodily and post-stroke symptoms regardless of level of disability. They also frequently described a positive affect and optimism towards recovery. Some, especially those with ‘good’ self-rated health and significant disability also found meaning from their stroke, reporting a spiritual outlook including practicing daily gratitude and acceptance of limitations. Individuals with minimal disability reporting ‘fair’ or ‘poor’ self-rated health on the other hand frequently referred to their post-stroke physical symptoms and comorbidities and indicated anxiety about future recovery. These differences in psychological outlook clustered with differences in perception of relational and social context including support offered by family and healthcare professionals.

**Conclusions:**

The disability paradox may be illuminated by patterns of individual attributes and relational dynamics observed among stroke survivors. Harnessing these wider understandings can inform new models of post-stroke care for evaluation.

**Supplementary Information:**

The online version contains supplementary material available at 10.1186/s12883-021-02338-x.

## Background

It is often assumed by those who are able-bodied that people with physical disability lead lives of lower quality [1]. However, many living with disability including many stroke survivors rate their own quality of life and health as good [2–4]. In fact, in a previous study we found over 70% of stroke survivors, most with some level of residual disability, to report ‘good’ or even ‘excellent’ self-rated health (SRH) [2] - a summary measure of subjective health perception that predicts the course of disability and institutionalisation in older people, as well as functional outcome and return to work in stroke survivors [5–10].

This phenomenon, where there is an apparent disconnect between a person’s observed level of disability and their own self-ratings of their quality of life or health, has been called the “disability paradox” (Albrecht and Devlieger, 1999) [11]. From interviews with 153 individuals with a range of physical disabilities, Albrecht and Devlieger reported 54.3% of respondents with moderate to serious disabilities to have an excellent or good quality of life. As Krahn points out even people with significant spinal cord injuries, visual loss or intellectual disability can become athletes, have an apparent good quality of life, and live normal life-spans, supporting the “disability paradox” in the real world as well as in self-reports [11, 12]. Explanations for the paradox have therefore pointed to the limitation of medical models of health and instead highlighted the relevance of psychosocial explanations and of feelings of control over their lives in those with disability [11, 13]. Albrecht and Devlieger have indeed identified a number of attributes of the ‘body’ - physical function dimensions’, ‘mind’ - rational and intellectual capacities’ and ‘spirit’ - recognition that the self is part of a higher order of the universe/higher being or having a purpose in life beyond the self’, that together with environmental context could explain the paradox [11].

In a previous qualitative study, we explored what defines health for stroke survivors in a heterogeneous group of participants and identified a number of influences that play a role in their subjective health experience [14]. To understand now the paradox of subjective perception of good health despite disability in some stroke survivors and to inform the development of new models of post-stroke care, we turn to investigating in this paper the specific relationship of self-rated health with disability in this group. We specifically address using data analysis in smaller groups of our stroke survivors from the larger cohort, why some have levels of self-rated health concordant with their disability levels while some with none or only minimal post-stroke disability see themselves only in fair or poor health and yet others rate themselves as healthy in spite of significant objective post-stroke disability - “disability paradox”.

## Methods

This is a separate analysis of data collected in a previous study [14]. We used qualitative interviews to explore what factors respondents perceived contributed to their subjective health experience. The study comprised 28 interviews conducted 4 to 6 months after stroke with full details described elsewhere [14].

### Recruitment and sampling

Ethics approval for the study was obtained from the National Health Service (NHS) East of England – Norfolk Regional Ethics Committee (REC) (ref 11/EE/0108). Potential participants were identified from a rehabilitation stroke unit at Cambridge University NHS Foundation Trust Hospital and a follow-up outpatient clinic and approached face to face by a stroke consultant or a specialist stroke nurse who was familiar with the patient. Potential participants who were deemed medically and ethically incapable of consent including due to significant cognitive deficit were not invited to participate under the guidance of the specialist consultant overseeing the study. Written informed consent was obtained from all eligible participants before interview for use of their data in synthesis of qualitative research. Methods including characteristics of those interviewed have been described previously [14]. A convenience sampling approach was used for recruitment and where possible participants were recruited from a range of ages and levels of disability. We excluded stroke survivors with severe clinical aphasia and cognitive deficits (clinically assessed as a Mini Mental State Examination score of less than 20) [15], and those who did not speak English.

### Data collection

Of 45 stroke survivors approached, 28 agreed to participate. Measures were taken by researchers NM and LL and included age, gender, socioeconomic status; Index of Multiple Deprivation [16], physical disability levels; Modified Barthel Index of Activities of Daily Living [17], number of physical comorbidities, and mental health status; and the Hospital Anxiety and Depression Scale [18]. Participants were asked the single self-rated health question: “How would you rate your general health?” with 5-point Likert scale responses: ‘very poor’, ‘poor’, ‘fair’, ‘good’ and ‘excellent’.

Interviews were semi-structured, and were carried out by NM, LL, and ES at the participants home and lasted between 45 to 80 min. Carers and spouses were present in around one third of interviews. However any comments made by carers or spouses were not considered in the analysis of data. Field notes were taken where relevant to corroborate and enhance interview findings. NM and LL are female General Practitioners with medical qualifications and a background in community-based research, and ES is a male physiotherapist and social scientist with a background in stroke research and extensive experience in qualitative research. NM and ES hold PhD degrees. NM and LL had previously each met some of the participants during the recruitment process and during administration of questionnaires, while ES met the participants for the first time at interview. Participants were aware of the interviewers clinical and research backgrounds. Interviewers did not report to participants any personal biases with respect to the research being carried out outside of clinical and research interest in helping stroke survivors with their rehabilitation. Interviewers asked participants how they would describe their present health since the stroke, followed by further questions form the interview prompt derived from previous consultation with patient volunteers [14]. Based on the responses from interviewees we were able to explore why some stroke survivors with disability rated their health as poor and others as good. All interviews were audio-recorded, transcribed verbatim and then stored, managed and coded in NVivo (Version 9.0) Computer Aided Qualitative Data Analysis Software.

### Data analysis

For this analysis, 15 stroke survivors in which there was a mismatch between levels of self-rated health and level of physical disability as measured by the Modified Barthel Index: [17] i.e. participants with (i) better self-rated health (‘excellent’ and ‘good’) and significant physical disability (Barthel Index less than or equal to 17) and (ii) poorer (‘fair’ or ‘poor’) self-rated health and assessed as minimally disabled (Barthel Index greater than or equal to 18) were compared to each other. They were also compared to a control group of 13 participants (9 with ‘good’ and 4 with ‘excellent’ self-rated health) whose assessments of subjective health were concordant with the physical outcome from their stroke (i.e they had minimal levels of post-stroke disability). There were no participants with poor self-rated health and significant physical disability in our sample.

Transcripts were read and re-read and coded for themes emerging from the data using a thematic analysis approach and the constant comparative method [19] by NM and LL with input from ES and CM until data saturation was reached as determined by discussion between NM, LL, ES and CM. Data were organised using matrices to facilitate comparisons between participants in the three groups of stroke survivors. Identified themes were then categorised using the broader themes identified by Albrecht and Devlieger as contributing to the ‘disability paradox’ in the area of quality of life: body, mind, spirit and the environment [11].

## Results

Participants in the study were aged 47–86 years, of whom 19 were men and 9 women. Table 1 (a)(b) and (c) show the characteristics of study participants and Table 2 shows these data for the three study groups.

**Table 1:** Socio-demographic characteristics and psychological status of study participants

Study ID	Sex	Age	Comorbidities Number	Index of Multiple Deprivation (IMD) †	Depression Score (HADS-D) ‡	Anxiety Score (HADS-A) §
**(a) Better self-rated health (‘excellent’ and ‘good’) and significant physical disability (Barthel’s Index = ≤17) (*n*** **= 8)**						
*Excellent*						
A	M	60–64	2	2	4	8
B	M	60–64	3	1	8	12
*Good*						
C	F	> 85	3	3	6	15
D	F	60–64	4	4	4	3
E	M	> 85	3	1	2	4
F	M	80–84	3	1	9	9
G	M	75–79	2	1	1	4
H	M	> 85	2	1	6	15
**(b) Poorer self-rated health (‘poor’ and ‘fair’) and minimal physical disability (Barthel’s Index ≥ =18) (*n =*** **7)**						
*Fair*						
I	M	70–74	2	1	6	10
J	M	65–69	1	3	7	0
K	M	75–79	2	4	3	10
L	F	50–54	3	3	8	13
M	M	45–50	1	1	3	7
N	F	65–69	4	2	2	5
*Poor*						
O	F	60–64	2	1	11	13
**(c) Better self-rated health (‘excellent’ and ‘good’) and minimal physical disability (Barthel’s Index > =18) (*n =*** **13)**						
*Excellent*						
P	M	70–74	2	2	0	0
Q	M	75–79	2	1	0	0
R	M	70–74	3	1	1	5
S	M	70–74	1	4	2	1
*Good*						
T	M	65–69	3	1	6	7
U	F	80–84	2	2	2	0
V	M	55–59	1	2	2	5
W	F	45–49	4	2	5	7
X	M	60–65	3	1	2	6
Y	F	70–75	3	1	3	7
Z	F	75–79	2	2	7	7
AA	M	65–69	2	1	3	4
BB	M	70–74	2	2	3	7

**Table 2:** Characteristics of study participants with stroke by self-rated health (SRH) and disability level groups

	All (*n* = 28)		‘Excellent’ and ‘good’ SRH with significant disability ^a^ (*n* = 8)		‘Fair’ and ‘poor’ SRH with minimal disability ^a^ (*n* = 7)		‘Excellent’ and ‘good’ SRH with minimal disability ^a^ (*n* = 13) N %	
	N	%	N	%	N	%		
Sex								
Female	9	32.2	2	25.0	3	42.8	4	30.8
Male	19	67.8	6	75.0	4	57.2	9	69.2
Age (years)								
> =85	3	10.7	3	37.5	0	0	0	0
65–84	20	71.4	5	62.5	5	71.4	10	76.8
= < 64	5	17.9	0	0	2	28.6	3	23.2
IMD by quintiles^b^								
1st & 2nd	22	78.6	6	75.0	4	57.2	12	92.3
3rd, 4th & 5th	6	21.4	2	25.0	3	42.8	1	7.6
Co-morbidities (number)								
One	4	14.3	0	0	2	28.6	2	15.4
Two or more	24	85.7	8	100	5	71.4	11	84.6
Depression ^c^								
Severe (HADS > 11)	1	3.6	0	0	1	14.3	0	0
Moderate (HADS-D 7–10)	3	10.7	2	25.0	1	14.3	0	0
Anxiety ^d^								
Severe (HADS-A > 11)	4	14.3	2	25.0	2	28.6	0	0
Moderate (HADS-A 7–10)	6	21.4	3	37.5	2	28.6	1	8.0

^a^BI Barthel Index (significant disability BI = < 17, minimal disability BI> = 18)

^b^IMD Index of Multiple Deprivation 1 = top 5 = lowest

^c^HADS-D Hospital and Anxiety Depression Scale- Depression

^d^HADS-A Hospital and Anxiety Depression Scale- Anxiety

Below we report key findings where differences were observed between subgroups of stroke survivors with illustrative quotes from participants’ to draw out examples of the disability paradox. All stroke survivors regardless of their perceived level of self-rated health discussed their health in the context of their current physical function and limitations, which included difficulties with ambulation, activities of daily living and speech. In the quotations below, pseudonyms are provided to protect the anonymity of participants.

### (i) Better (‘excellent’ and ‘good’) self-rated health with significant physical disability *(N* = 8).

#### Body

Stroke survivors in this group reported substantial focus on their physical rehabilitation since their stroke. They set themselves detailed goals, took proactive steps towards their rehabilitation, made regular time, carefully practiced and created their own exercises to progress their rehabilitation. Their accounts reflected a strong desire and expectation to return to a sense of normality and a refusal to be defined by their stroke. Their responses also reflected resilience, being content to make small steps of daily progress and meet setbacks with determination until they reached their goal. For example:

*“Well I’ve got to look after myself, naturally. Just keep pushing on, try and get back to reality as best as I can really... I’m going to keep going and keep trying different things so I can get back doing everything I wanted to, you know” (Mr. A, 60–64, excellent SRH).*

*“I aim to do one more thing each day … If I do that, ‘ooh, that’s better than yesterday. Good.’ Things, little little things like that.” (Mrs. C, 85–89, good SRH).*

Being independent and resolving to carry out everyday tasks and activities on their own without relying on others contributed to a sense of normality and a greater confidence in achieving their physical rehabilitation goals:

*“I come down and I said to her ‘I’ve just had a bath’ and she said ‘who put the seat in?’ I said ‘nobody’, I said ‘I didn’t put it in’, I said ‘I got in myself’, she went ‘what’? I said ‘I got in and out the bath myself’, she was ‘blimey’.” (Mrs. D, 60–64, good SRH).*

#### Mind

This group did not generally report feeling low in mood in the face of significant disability, although one with ‘excellent’ subjective health reported having received antidepressant medication immediately after his stroke and another with ‘good’ subjective health had moderate depression on testing. These survivors, especially the two with ‘excellent’ self-rated health, frequently spoke of even feeling happy and positive with regards to their present circumstances and particularly with their progress in rehabilitation. Many held the belief that they were overcoming their stroke and commonly voiced optimism and a positive outlook with respect to their future recovery. For example:

*“Feel like I’m winning all the time … Yeah, winning over the stroke, yeah … That’s why I want to see progress. It’ll come, I believe it will come.” (Mr. A, 60–64, excellent SRH).*

*“I’m quite optimistic about the future … I think well things will get a bit better, yeah. I’m normally optimistic every day.” (Mr. E, 85–89, good SRH).*

At the same time, these survivors were willing to face uncertainty regarding their future and showed room for flexibility in their accounts for reassessing their future capacity for progress or the possibility of stroke recurrence. One survivor with ‘excellent’ self-rated health articulated this attitude of willingness to accept his future whatever that may be:

*“I think the future comes anyway, you know, it’s...what will be will be, you know. I might live 20 years, I might live 10, you know … maybe I say goodbye to strokes, maybe I’m alright (laughs) … And I hope to get back to driving. If not, I, you know, make contingency plans, you know.” (Mr. B, 60-64, excellent SRH)*

#### Spirit

This group most often had a philosophical attitude towards their stroke and strove to derive meaning for their disability and for life in general from the event. Their philosophical outlook included frequently reporting the acceptance of and adaptation to their physical limitations and circumstances and a perception that more trivial problems of life no longer mattered in the face of having suffered a stroke. A couple of these survivors considered their stroke as not having been a bad thing, even describing it as having been for the best. Most also reported a sense of daily gratitude for having survived their stroke and a feeling of being lucky to be alive:

*“I know all this has happened, looking back over our lives together … Things have happened for the best always … Oh yes, every morning I wake up and thank God for the gift of a new day.” (Mrs. C, 85-89, good SRH)*

The stroke made some survivors more people-minded, less judgmental and more patient and appreciative of others, and led them to having a spiritual and altruistic outlook on their relationships to others including the desire to try to help others despite their disability. As one man said:

*“You suddenly realise that you’re not an island, you’re one of very many and you need, you need others as they need you.” (Mr. E, 85-89, good SRH)*

Three reported drawing on a power greater than themselves including their faith in God to help them overcome their fears of the future and with their recovery from their stroke. Two of them especially felt that having had a stroke had made them even stronger in their faith. One of them, Mrs. C continued to say:

*“Well, the comfort of being able to talk to Him and tell Him all of my thoughts and worries and cares. That’s it in a nutshell … I never had any moments of doubt that I wouldn’t get better, and of course eventually I did … Because I had someone to talk to that understood. It’s not a new thing with me. I’ve always had a strong belief … It has strengthened … Because I’ve got over the difficult situation through faith … Yeah, that’s what brought me through.” (Mrs C, 85-89, good SRH)*

Another stroke survivor explained how reliance on God worked together with his own determination in the path of his recovery:

*“And, you know, miracles are not something that he comes down and gives you a new hand, it’s just something you’ve got to do yourself, you’ve got to, it’s no good relying only on God, you’ve got to say ‘I will do something about it’ and you’ve got to try yourself.” (Mr. E, 85-89, good SRH)*

Two survivors with excellent self rated health in contrast saw the source of their strength to lie in their own personal ability to cope and the cause of their stroke as likely due to lifestyle factors under their own control. These survivors also did not report the stroke as having changed them much, saying they had always been optimistic and positive.

#### Environment

Stroke survivors in this group were mostly content with their current circumstances and reported having most of the things they needed in their environment to help them cope with their stroke including financial resources and family and friends to help them feel positive and to give them positive encouragement to face their physical disabilities and to persist in their rehabilitation. For example, one woman said:

*“Well, really, it’s very wonderful. People, friends, carers coming in all day. I love to see them because we have some lovely chats ...Well, to tell you the truth, I’m lapping it up, all this kindness, giving me strength to go on ... Because they keep saying, ‘ooh, you couldn’t do that the other day. You’re getting on every day a bit better’.” (Mrs. C, 85-89, good SRH)*

Such stroke survivors also reported mainly positive perceptions of the support provided by health professionals and particularly rehabilitation therapists following their stroke. This often related to recollections of positive interactions and encouragement in relation to their progress. For example, one man explained how the physiotherapist’s encouragement inspired him to work harder, also saying:

*“Then I had the physios come in, stroke team come in, and they’ve been absolutely brilliant … Yeah, she inspired me to keep going. She’s brilliant … ‘Brilliant’, she says. She says I’ve been one of the star pupils, yeah.” (Mr. A, 60-64, excellent SRH)*

### (ii) Poorer (‘fair’ and ‘poor’) self-rated health with minimal physical disability (*N* = 7)

#### Body

Most participants in this group spent less time in their interviews focussing on their physical rehabilitation. For a majority, rehabilitation effort was hard work. For example:

*“Because my life is … is turned off in a way, but it isn’t, but I’m determined to do it, to do some work … Well just work hard and … and … yes, just work hard.” (Mr. I, 70-74, fair SRH)*

This group also tended to have symptoms including pain, lethargy and other comorbidities. Lack of physical energy and tiredness were particularly prominent, and this impacted their desire to work on their rehabilitation or to carry out some of their daily activities. For example:

*“I don’t want to do this anymore, I have enough … I get tired. I get tired and I think, ‘O God, don’t overdo it. I’m overdoing it.’” (Mrs. O, 60-64, poor SRH)*

#### Mind

This group tended to report negative mental effects of their stroke including low mood, irritability, anxiety and difficulties with coping. Several reported physical or mental inactivity during their day and did not attempt to schedule activities due to lack of motivation. For example, one man commented:

*“I don’t know, because I don’t really do nothing when I get up, if you know what I mean.” (Mr. J, 65-69, fair SRH)*

Furthermore, a minority reported that the stroke had changed them for the worse, especially in becoming more irritable and impatient with others:

*“Erm, irritable I think, I found that the little things used to get on my nerve ... I mean I’ve even shouted at me wife and I’d never, ever done that in forty years. ... Well yes, yeah, because I’ve never been like that before, it’s only since the stroke that I’ve started letting things build up on top of me.” (Mr. K, 75–79, fair SRH).*

Attitudes to recovery and expectations for the future among such stroke survivors were generally guarded. While one reported optimism about recovery, several others appeared to be only hoping rather than expecting that they would be able to get back to previous levels of independence. They often felt anxious and found it difficult to cope with the uncertainty regarding their future, especially with respect to the possibility of a fall or a recurrence of their stroke:

*“I think it gave me some fear for the future now. Just fear that if that came on so innocently, that maybe I’ll be driving and something would happen … I feel like I’d better hurry up and see what I’m going to see in the world … I want to continue to be able to do things while I’m healthy and realise that at any time, I could have another stroke and I may not be able to walk or dance ... And it’s happened and I know these little vessels that I’ve got are all affected now, it’s a little bit of a time-bomb waiting to happen.” (Mrs. L, female, 50-54, fair SRH)*

#### Spirit

All seven stroke survivors in this group struggled with the acceptance of their stroke. Five out of the seven reported difficulties accepting their current level of disabilities including their inability to carry out usual activities prior to stroke including sport and social activities. One survivor found it very difficult to accept her stroke and said she did not find “any good in it at all” since the stroke had affected her outlook on what she could achieve in life. None expressed any particular philosophical perspectives on why they had suffered a stroke, having not given much thought to it. If they had, they frequently articulated a ‘why me’ attitude or felt that their stroke was a result of bad luck or part of the ‘ups and downs’ of life:

*“I don’t know, until I talked to you, I never really thought hard about it, I never really thought about it, but probably (laughs) … So you think these things are going to happen because they’re just part of … part of life … part of life, right … and death, yeah.” (Mrs. L, 50-54, fair SRH)*

None reported having faith in God or an external focus to rely on for their journey of recovery:

*“I didn’t have a lot of religious beliefs or stronger religious beliefs after the stroke than I did before, it was the same. And so I know these things are going to happen and I don’t feel the need to all of a sudden rush off to church and start praying. I don’t sit down and say ‘God, please help me’ or anything like that.” (Mrs. O, 60-64, poor SRH)*

#### Environment

The majority of stroke survivors in this group commonly reported a loss of role and status in society, such as in being a bread-winner or carer, and identified financial and other struggles such as challenges with work and maintaining social activities and relationships, which were particularly evident among the men in the group. For example, one man who previously worked as an electrician described not going to work to be “as if his life had been turned off” and another articulated how the impact of the stroke had meant that he had no longer been able to assist his disabled wife with daily tasks as he desired, which made him feel “useless” since this had until the time of his stroke been a main focus of his life.

Most reported feeling dependent on their families for support with several saying that they could not have done without their partner or children supporting them through the stroke. However, while these stroke survivors said that they were likely to rely on family members for practical help such as with shopping and outings and emotional help to uplift their mood, some reported difficult relationships with family members. One female survivor spoke about the lack of help she had received from her husband and adult children who expected her to carry out her household duties after the stroke as she had done prior. Another said she sometimes got into arguments with her partner over doing her exercises, since she felt he was pushing her too hard:

*“Well, it’s up to you, you get on and do it, you know, you do more exercise. It made me crabby. So, it caused not arguments, but … yes, arguments I suppose.” (Mrs. O, 60-64, poor SRH)*

On the other hand, a couple of survivors in the group reported family members wrapping them up metaphorically in cotton wool and discouraging them from overexerting or tiring themselves, which may have inadvertently hindered their early rehabilitation. As one survivor with ‘fair’ self-rated health said regarding his spouse:

*“She (wife) won’t let me do something that she knows I can’t do and if I’m trying to do something then she’ll say ‘stop, leave it, leave it alone now, have another go later’ but she don’t turn around and say ‘oh go on, get on with it’” (Mr. K, 75-79, fair SRH)*

### (iii) Better (‘excellent’ and ‘good’) self-rated health with minimal physical disability in comparison to groups (i) and (ii) (***N*** = 13)

#### Body

Stroke survivors in this group focused on the process of their physical recovery and on any remaining physical limitations. They did not frequently report on bodily symptoms and gave attention to keeping their body healthy mostly through lifestyle including their diet, exercise, smoking and alcohol intake:

*“In one way it’s made me change my lifestyle drastically which is a good thing, I’m probably a better person to know now, having stopped smoking, like people say ‘oh you haven’t?'” (Mr P, 60-64, good SRH).*

They were more determined and better able to make lifestyle changes compared to their counterparts with significant physical disability due to the reduced demands from their physical rehabilitation and to a greater time and physical capacity to focus on behavioural changes. Improving their lifestyle may have also improved these survivors’ perceptions of their health.

#### Mind

Stroke survivors in this group had similar mental traits to those with better subjective health and significant physical disability including a positive and optimistic outlook on life despite having recently suffered a stroke. These survivors focused on gains in their recovery and were prepared to move on with their lives, attributing much of their recovery to their own independence and determination. These survivors, similar to their counterparts with significant physical limitations and unlike those with poorer self-rated health, did not often describe negative mental symptoms such as anxiety and worry over their future and had mainly positive views of their future recovery. A few saw themselves mentally as youthful and energetic:

*“I don’t want to be old, I hate being old! Well, being with you young people...I think their attitudes are all different, much more refreshing than older people, I think … . Yeah and having young friends, I think, is another thing that keeps you going. … . They make you go out. They make you do the things that they do at 50” (Mr P, 70-74, excellent SRH)*

#### Spirit

Most survivors in this group reported attitudes of acceptance and gratitude in their post-stroke lives, for having recovered with few physical limitations and for their return to near normality. They less frequently reported pondering the meaning of their stroke compared to those with greater disability, or beliefs in God.

#### Environment

All stroke survivors in this group reported a good quality of support from their families and friends. They tended not to report negative socioeconomic circumstances and were more likely to report having returned to work and to having maintained their pre-stroke lifestyle after the stroke. One said:

*“I do a lot of walking. I go and visit different towns just to get out and do something really... I went on holiday in February, I went to the Gambia for 12 days. I’m going off to Singapore and to Borneo in September for 17 days … Well, I don’t lack anything that I feel that I need. I’m not short of a few shillings, I have lots of people around me that are great friends, I have a good life, I can do whatever I want to do.” (Mr P, 70-74, excellent SRH)*

#### Summary

In summary, stroke survivors drew on psychological, social and spiritual resources to enable them to maintain a sense of health and wellbeing in the context of the physical impacts of stroke. Those with minimal disabilities and better self-rated health responded differently to those with poorer self rated health with similar levels of physical disability. This suggests that the role of disability in self-rated health perception is influenced by context and individual traits beyond functional limitations.

Table 3 summarises a number of important differences found in our analysis between stroke survivors who showed discordant self-rated health and disability levels in the areas of ‘body’, ‘mind’, ‘spirit’ and ‘environment’ as per Albrecht and Deveglier’s ‘disability paradox’ paradigm.

**Table 3:** Differences found in identified themes^a^ in participants with stroke with discordant levels of self-rated health (SRH) and physical disability

	Excellent and Good Self-rated Health Significant Physical Disability	Fair and Poor Self-rated Health Minimal Physical Disability
**Body**	These stroke survivors reported a sense of agency over their bodies. They set goals and were determined in the rehabilitation of their bodies and in improving their physical lifestyles through diet and exercise. They did not wish to be defined by their stroke and desired to return to normality of physical functioning.	These stroke survivors frequently reported their physical symptoms of pain and fatigue, saw their bodies more negatively and as aged, found rehabilitation hard work and were less focused on making necessary changes to improve their bodily health.
**Mind**	These stroke survivors, in particular the two with ‘excellent’ self-rated health reported being happy and optimistic about their progress in rehabilitation and their future recovery, as well as having a resilient attitude to setbacks together with the willingness to accept uncertainties about their future.	These stroke survivors often reported poor motivation, low mood and anxiety, and expressed fear-based and negative cognitions regarding the potential for recovery, stroke recurrence, and of decline of health with ageing.
**Spirit**	These stroke survivors reported a highly independent attitude when thinking about their recovery, drawing predominantly on their own personal strength in the process of rehabilitation. However, number of the stroke survivors with ‘good’ self-rated health but significant disability, relied on God and drew strength from their faith for their rehabilitation, intentionally practiced gratitude and acceptance of their limitations and exhibited altruistic characteristics, looking beyond themselves and their own situation to consider befriending and helping others.	These stroke survivors mostly portrayed less of a philosophical attitude towards their stroke and appeared to struggle to find meaning from their stroke.
**Environment**	These stroke survivors mostly enjoyed better socioeconomic status and access to financial resources to moderate the burden of ill-health and disability. They mostly reported supportive relationships with family and therapists who were encouraging.	These stroke survivors reported adverse post-stroke social circumstances such as loss of family and societal roles including with employment and finances. A few of these survivors reported dysfunctional families. Some reported family members that discouraged them from pushing themselves to complete rehabilitation tasks or activities of daily living on their own for fear of them becoming over-tired or having a setback.

^a^Themes divided into areas of ‘body’, ‘mind’,‘spirit’ and ‘environment’ as per Albrecht and Devlieger’s ‘disability paradox’ [11]

## Discussion

**Fig. 1:**
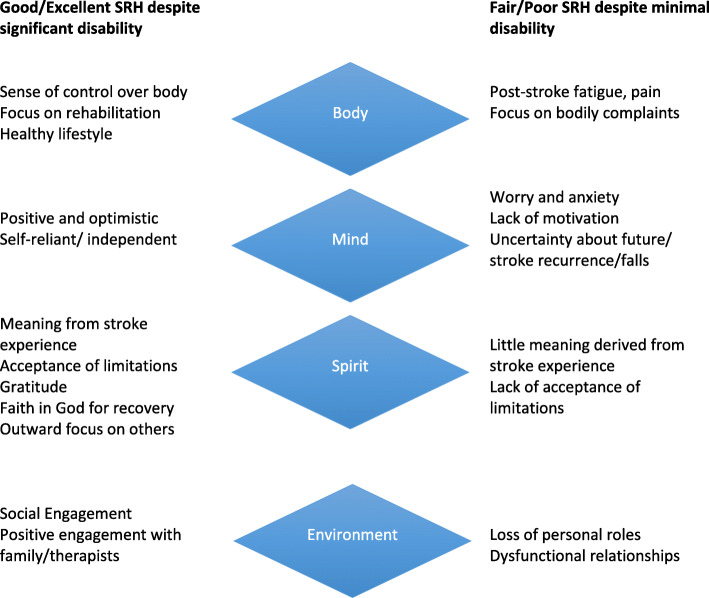
Body, mind, spirit and environmental influences on perceived self-rated health (SRH) in participants with discordant levels of SRH and physical disability

In this study we draw attention to possible explanations for the ‘disability paradox’ among people living with stroke (Fig. 1). A number of specific psychosocial resources in stroke survivors with better self-rated health in our study may have mitigated against the negative effects of significant disability on health perceptions and allowed such survivors to maintain a sense of wellness in the face of disability. While these were shared amongst those with all levels of disability, these resources gained particular importance in the context of rising to the challenges of rehabilitation in those with significant physical limitations. Outstanding among these resources were reports of a positive outlook and optimism regarding progress in rehabilitation and the future outcome of stroke. In addition, those with better self-reported health tended to describe a sense of control and strong faith in either their own ability to overcome the challenges of their stroke, or faith in an omnipotent source outside of themselves to draw upon. They made positive meaning out of their stroke and were more likely to adapt and accept any functional limitations. In contrast, those with lower perceptions of self-reported health did not take meaning from their stroke, had a negative outlook on the future, focussed on the self and on bodily limitations, pain and comorbidities. Environmental context and resources, including finance and social resources and support appeared to shape the dissonance in stroke survivors in our study between subjective and objective indicators of health. Good quality of social resources available to stroke survivors with better self-rated health contrasted to the sometimes challenging contextual circumstances including dysfunctional family dynamics that may have contributed to a sense of helplessness towards stroke rehabilitation in those with poorer subjective health.

Albrecht and Devlieger suggest that people with disability who report poorer quality of life relate this to the experience and loneliness of having pain, fatigue and loss of control, while those who report better quality of life attribute this to feelings of control over their bodies, minds, and lives [11]. Similarly, it has been proposed that self-ratings among those with poorer self-rated health are largely a reflection of the physical experience of ill-health including pain and medication burden, while in those with better self-rated health, these perceptions may be buffered by contextual factors including lifestyle and psychosocial resources [20–25]. Stroke survivors who viewed themselves as healthy in our study showed a combination of traits and resources consistent with notions of resilience, agency and sense of control in the face of disability, as well as a realistic optimism towards their future moderated by an ability to take life as it comes. As portrayed by Gold in his study of successful rehabilitation, these stroke survivors were ‘optimistic but firm’, [26] characteristics of survivors that lead to improved levels of adjustment and the ability to ‘bounce back’ following a stroke [27]. Fellinghauer et al. and others suggest that positive environmental factors such as social supports that minimise impact on societal involvement may mean that physical impairments do not lead to expected reductions in quality of life and subjective health perception in those with disability [11, 13, 28–33]. The positive resources seen in stroke survivors with better self-rated health in our study were frequently reinforced by their social supports and positive interactions and encouragement from family and therapists who did not cast them into a ‘sick role’ [34, 35]. These interactions may have led the stroke survivor to either an upward or downward spiral of recovery and health, ‘wellness’ or ‘illness’ in the face of disability [36, 37].

Our findings support the value of a wider biopsychosocial model in which the dynamic inter-relationship between the patients` own psychosocial resources, and family, carer and therapists input could lead in the face of disability to a view of wellbeing despite the challenges of rehabilitation. These findings also argue for humility in applying the medical model alone in stroke care and inclusion of a wider salutogenic model [38]. Our study provides health professionals with insights that help sensitise them to the potential of each stroke survivor as an active agent exercising control over their life and enables them to offer support that builds on the individuals views and existing coping strategies, drawing from the strengths identified in those who have been able to maintain their sense of wellness in the face of disability. At the individual level these emphasise the relevance of responding to the ways in which the stroke survivor and their families make sense of the survivor’s disability and health in the weeks and months following stroke, while at a group level they draw attention to approaches that encourage a sense of ‘wellness’ rather than ‘illness’ in survivors. Specific approaches in which these findings could be incorporated include the sharing of positive stories from those who have maintained a sense of normality in their journey of stroke through peer support groups and social media and psycho-education, including for families and therapists. Training for stroke survivors in positivity, realistic optimism and resilience including strategies such as daily gratitude and acceptance [39–45], attitudes found in survivors with better self-rated health, require further study as potential means of assisting survivors with poorer subjective health to maintain a sense of wellbeing despite disability.

### Limitations

We acknowledge the constructed nature of the qualitative interview where participants may have engaged in strategies to present the self in particular ways [46]. Those with severe stroke-related disabilities, including that of speech and cognition were excluded from our study, limiting conclusions to less affected participants. The Barthel’s Index may not be the best measure of objective disability because of ceiling effects [47]. Participants were from mainly white ethnicity and higher social class, limiting understanding to be gained from a wider social mix. We also note that there were more older stroke survivors in our better self-rated health and significant physical disability group, which may have biased responses since suffering a stroke may have had less psychosocial impact on these survivors with respect to occupational and financial status, and older people may have different expectations of their health compared to those who are younger [48]. We have also not addressed in our study the presence of neglect or anasognosia nor any neuroanatomical correlations to better self-rated health in our participants. The nature of qualitative methodology is to describe phenomena and relationships, not to test them statistically. Neither the strength of association, extent of moderation nor direction of causality can be established with the small number of participants in this analysis. We can, however, raise questions about the features we have observed to underlie the complexity of the relationship between physical disability and self-rated health and hypothesise regarding how the psychosocial resources identified might assist stroke survivors to feel better and live well despite disability.

## Conclusions

Disability does not equate to poor health [49], including among stroke survivors. Considering the experience of stroke survivors with good self-rated health in the face of significant disability is worthy of further study as a model for better post-stroke care with the intention of designing specific interventions that help ‘normalise’ life for survivors and could offer ways for them to make sense of their predicament and increase a sense of control, confidence, independence, autonomy and self-determination in rehabilitation.

## Supplementary Information

**Additional file 1.** Interview Guide for qualitative study

**Additional file 2.** COREQ checklist for qualitative study

## Data Availability

The datasets generated and/or analysed during the current study are not publicly available due participant consent not having been sought at the time for secondary data analysis outside the research team, or for the deposition of data in an archive.

## Abbreviations

SRH: Self-rated health
NHS: National Health Service
REC: Regional Ethics Committee
NIHR: National Institute for Health Research
CLAHRC: Collaboration for Leadership in Applied Health Research and Care
