# Supplementary material for: What underlies the difference between self-reported health and disability after stroke? A qualitative study in the UK
Source: BMC Neurol. 2021 Aug 13;21:315. doi: 10.1186/s12883-021-02338-x (PMC8362227; doi:10.1186/s12883-021-02338-x)
Supplement: Supplementary file 1 — Additional file 1. Interview Guide for qualitative study [file 12883_2021_2338_MOESM1_ESM.docx]

**Question Outline for Semi-Structured Interview**

**Core questions**

*Prompt questions if needed (only if interviewee needs prompting or core question needs exploring further)*

1. **Tell me about your experience of having had your stroke**

- *How is life different now from before? On a day to day basis?*
- *Can you describe any particularly good or bad experiences since having your stroke?*

**2) How do you feel in yourself?**

- *Has this changed since your stroke? If so, how?*
- *Is there anything that would help you feel better?*

**3) Tell me about your relationships with other people. How have any of these changed since your stroke?**

- *Family*
- *Friends*
- *Neighbours/colleagues*

- 1. **What do you think of your health? What makes you say this about your health?**
- *What things do you think have affected your health?*
- *Is there anything that you think would make you feel healthier?*
- *What do you seek medical help for? How could health services help you to feel better?*
- *Why do you think you had the stroke?*
- *Has the stroke made you think about your health in a different way?*

**5) What are your feelings about the future?**

- *How do you feel you will be in the future? Better or worse or the same and why?*
- *Do these feelings change?*
- *What do your family and health professionals think about how you are since your stroke and how do they think you’ll progress in the future?*
- *Is there anything that would make the future better?*
